# Acute Midgut Volvulus Due to Intestinal Malrotation and Coexisting Jejunal Diverticulosis in an Elderly Female

**DOI:** 10.7759/cureus.87198

**Published:** 2025-07-02

**Authors:** Akhil Chowdari Ganamani, Suresh Kumar Palanichamy, Chinni Vikram Asokan, Riddhima Dubhashi

**Affiliations:** 1 Surgical Gastroenterology, Sri Ramachandra Institute of Higher Education and Research, Chennai, IND

**Keywords:** acute midgut volvulus, adult intestinal malrotation, ladd's procedure, post-operative ileus, whirlpool sign

## Abstract

Midgut volvulus is an uncommon but critical complication arising from congenital intestinal malrotation. The condition typically presents during infancy, and adult-onset cases, particularly in elderly patients, are rare and often pose a diagnostic challenge due to their nonspecific and variable clinical presentation. We report the case of a mid-hexagenerian female with a known history of hypertension, aortic dissection with coronary artery disease, and chronic kidney disease, who presented with acute-onset colicky abdominal pain, bilious vomiting, and obstipation. Imaging revealed a 180-degree clockwise twisting of the distal ileal loop and the characteristic "whirlpool sign," indicative of volvulus. Emergency exploratory laparotomy confirmed midgut volvulus secondary to malrotation with Ladd's bands and an incidental finding of uninflamed broad-based jejunal diverticula. A Ladd's procedure was successfully performed to release adhesions and bands between the bowel and mesentery. Postoperative ileus complicated the patient's recovery but was resolved with conservative management.

In elderly adults, midgut volvulus secondary to intestinal malrotation should be considered in unexplained cases of intestinal obstruction. Progression to bowel ischemia can be prevented by early radiological evaluation and timely surgical management. Jejunal diverticulosis coexistence can be challenging when it is inflamed and perforated; however, in our case, it was a silent spectator not involved in the cause of volvulus.

## Introduction

Midgut volvulus represents a rare yet severe clinical disorder associated with congenital intestinal malrotation. Although primarily diagnosed in neonates and infants, there is a growing awareness of its occurrence among adults in current clinical practice. Adult-onset cases frequently manifest atypically, presenting considerable diagnostic difficulty, especially among elderly patients who might not exhibit classical volvulus signs. Unlike neonates, adults may exhibit chronic or intermittent symptoms such as nonspecific abdominal pain, episodic vomiting, and malnutrition, complicating the diagnostic process. Consequently, delays or inaccuracies in diagnosis are common, significantly elevating the risk of complications such as bowel ischemia and subsequent necrosis [1,2].

Pathophysiologically, the condition results from incomplete embryonic rotation and fixation of the midgut, leading to a narrow mesenteric base that predisposes the bowel to rotational torsion around the superior mesenteric artery (SMA). Such torsion jeopardizes vascular perfusion, precipitating acute obstruction [3]. While reported incidence rates for adult intestinal malrotation range between 0.2% and 0.5% with 15% of those cases exhibiting midgut volvulus, the true prevalence may be higher due to undiagnosed asymptomatic cases or those discovered incidentally during radiologic or surgical evaluation [4]. Due to the broader utilization of imaging techniques in emergency medicine, recent literature reports an increase in diagnostic rate [5]. The diagnostic imaging features in contrast-enhanced CT (CECT), like characteristic whirlpool sign, abnormal positioning of the duodenojejunal (DJ) junction, and inversion of SMA and superior mesenteric vein (SMV) relationship, as well as its higher sensitivity and less time- consuming nature, make it an effective preoperative imaging assessment in midgut volvulus [6,7].

Jejunal diverticulosis, characterized by the presence of sac-like mucosal outpouchings in the jejunum, is another rare entity, with a reported incidence of 0.5-2.3% and a predilection for older adults [8]. Most cases are asymptomatic and discovered incidentally during imaging or surgery. However, complicated diverticulosis can occasionally present with obstruction or perforation, though its coexistence with malrotation and midgut volvulus is exceedingly rare and typically incidental [8]. We describe a case of midgut volvulus secondary to undiagnosed intestinal malrotation with coexisting jejunal diverticulosis. This report underscores the importance of maintaining a high index of suspicion for malrotation in elderly patients presenting with volvulus as a cause of intestinal obstruction, as well as the relevance of recognizing incidental findings that may influence intraoperative decision-making.

## Case presentation

A 65-year-old female presented to the emergency room with a two-day history of abdominal pain. The pain was sudden in onset, colicky in nature, and localized to the epigastric and umbilical regions. She also reported three episodes of bilious vomiting of significant volume and had experienced obstipation for the past two days. There was no history of fever or similar episodes of colicky abdominal pain in the past. Her medical history included hypertension, chronic kidney disease, and coronary artery disease, for which she was receiving regular treatment with antihypertensives and aspirin. She had previously undergone a transvaginal hysterectomy 24 years earlier, a David procedure with coronary reimplantation for aortic dissection and complete heart block in 2015, and a permanent pacemaker implantation in 2022.

On examination, the patient appeared well-built with an ECOG 2 performance status. She was tachycardic, with a pulse of 110 bpm, blood pressure of 140/90 mmHg, and a pulse oximeter saturation of 96% on room air. She exhibited abdominal distension, although there were no signs of guarding or rigidity. Tinkling bowel sounds were heard on auscultation. Digital rectal examination showed a collapsed rectum. Laboratory investigations revealed a deranged kidney function test (Table 1). Other biochemical parameters and complete blood counts were within normal limits.

**Table 1:** Preoperative blood parameters BUN: blood urea nitrogen; INR: international normalized ratio

Parameter	Result	Reference range
Hemoglobin	11.3 gm/dL	12-15 gm/dL
Hematocrit	34.6%	36-46%
Total leukocyte counts	7750 cells/mm^3^	4000-11000 cells/mm^3^
Platelet count	2.15 lakhs/mm^3^	1.5-4.5 lakhs/mm^3^
Prothrombin time/INR	12 seconds/1.04	10.8-12.9 seconds
BUN	36 mg/dL	8-23 mg/dL
Creatinine	1.7 mg/dL	0.5-0.9 mg/dL
Albumin	4.3 g/dL	3.97-4.94 g/dL

**Figure 1:**
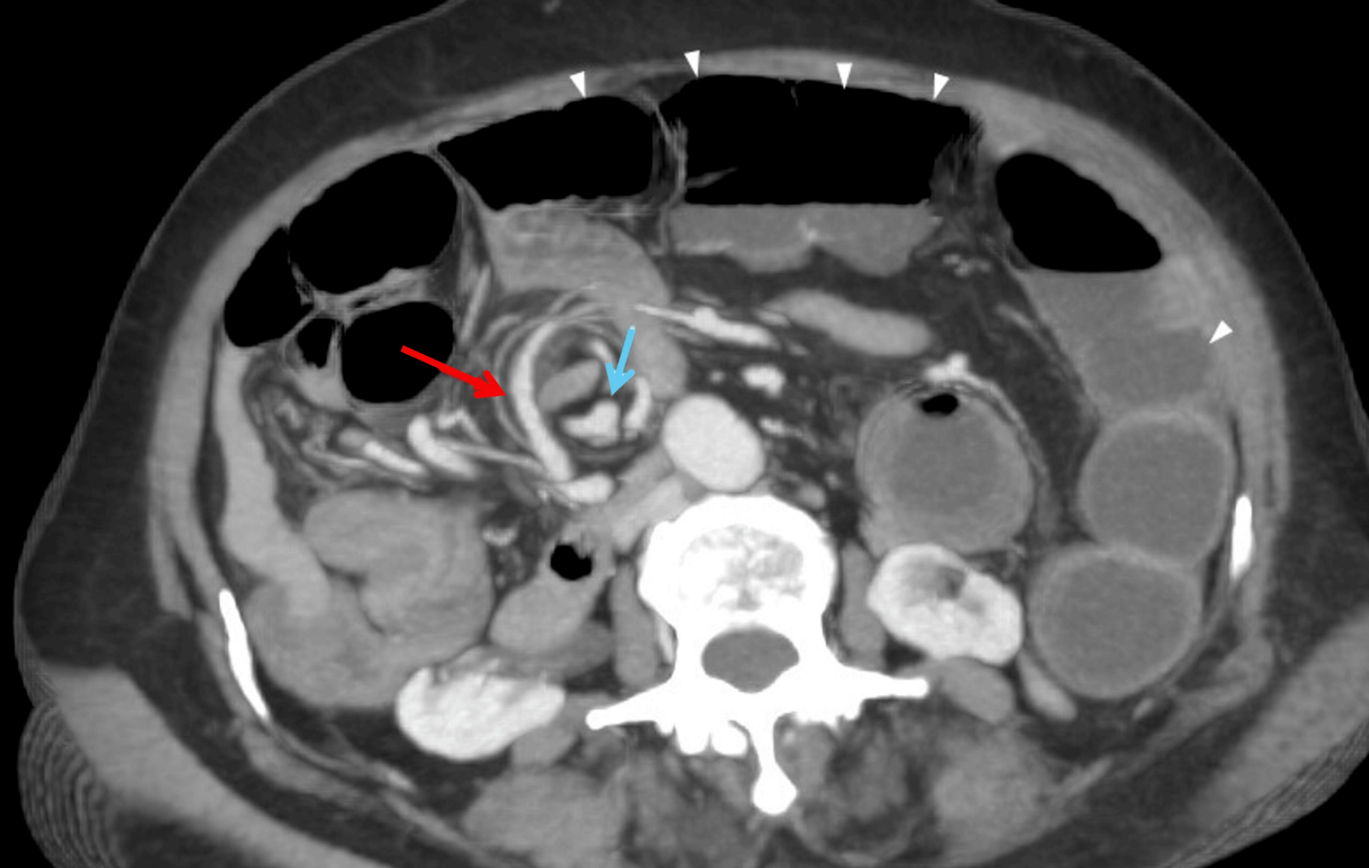
Venous phase of CT abdomen showing whirlpool sign with the SMA (red arrow) to the right of the SMV (blue arrow) and dilated small bowel loops (arrowheads) CT: computed tomography; SMA: superior mesenteric artery; SMV: superior mesenteric vein

**Figure 2:**
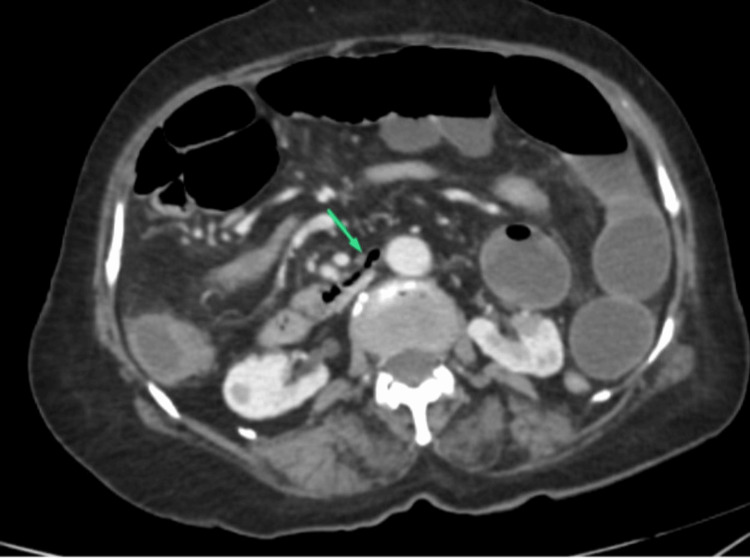
Duodenojejunal flexure (green arrow) to the right of the spine

**Figure 3:**
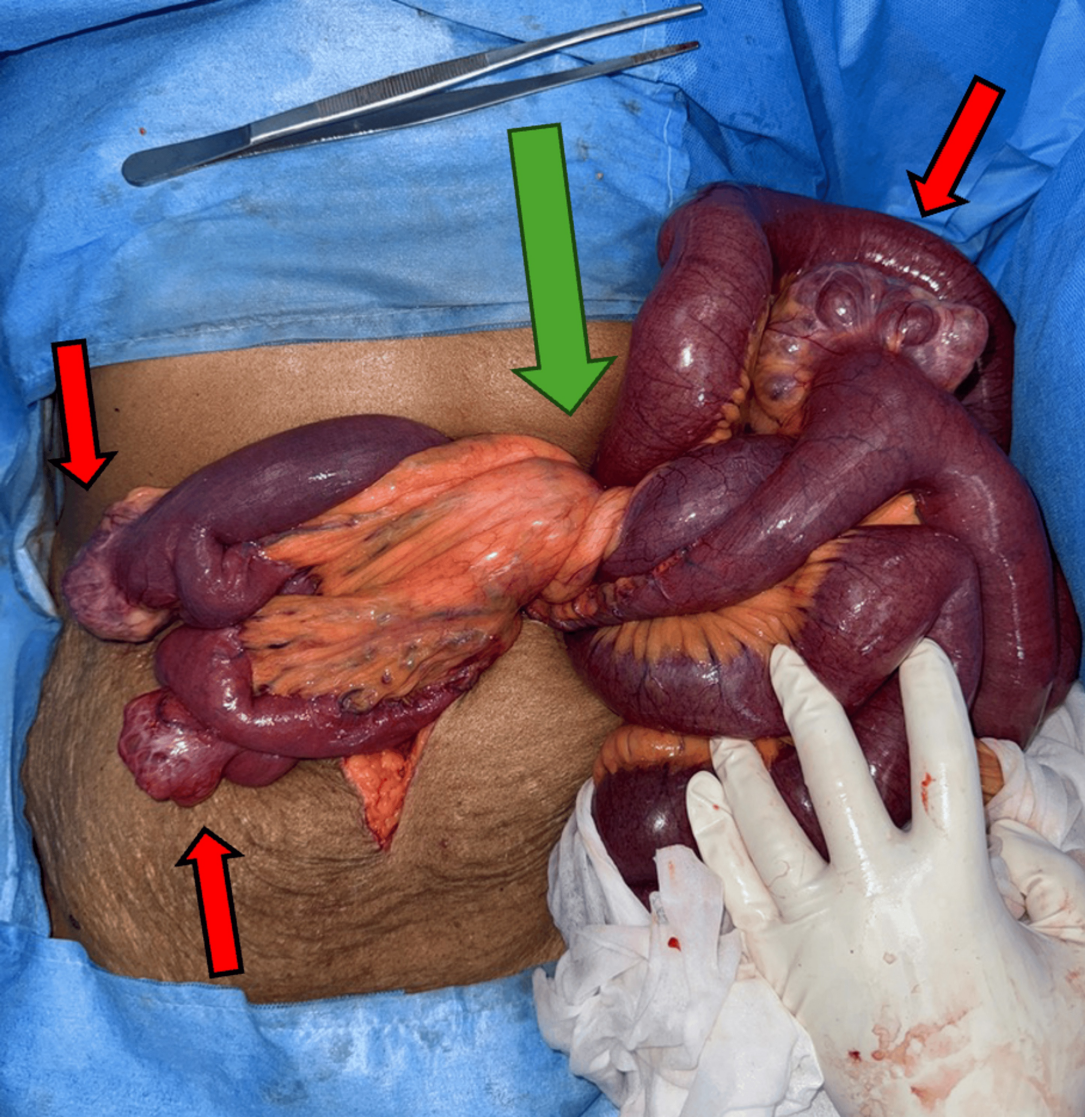
Intraoperative image of mesenteric volvulus (green arrow) with jejunal diverticulosis (red arrows)

Based on the clinical history of colicky abdominal pain associated with bilious vomiting, obstipation, and examination findings of abdominal distension and a collapsed rectum in digital rectal assessment, a provisional diagnosis of intestinal obstruction was made. Given the complicated cardiac history, acute mesenteric ischemia, probably due to embolus, was initially suspected. A CECT scan of the abdomen was performed. Imaging revealed clockwise twisting of the distal ileal loop with the mesentery, a 180-degree rotation of the SMV encircling the SMA - the characteristic "whirlpool sign" (Figure 1). The scan also showed dilated proximal ileal and jejunal loops with air-fluid levels and a transition point at the distal ileal loop within the volvulus. The bowel distal to the volvulus appeared collapsed. The SMV was tortuous and positioned to the left of the SMA, but there was no evidence of vascular compromise. Additional findings included a DJ flexure that failed to cross the midline, with the D3 and D4 segments of the duodenum located to the right of the spine (Figure 2). The large bowel was collapsed and in its normal anatomical position, with the transverse colon posterior to the small bowel (Figure 3). Bilateral simple renal cortical cysts were noted incidentally.

**Figure 4:**
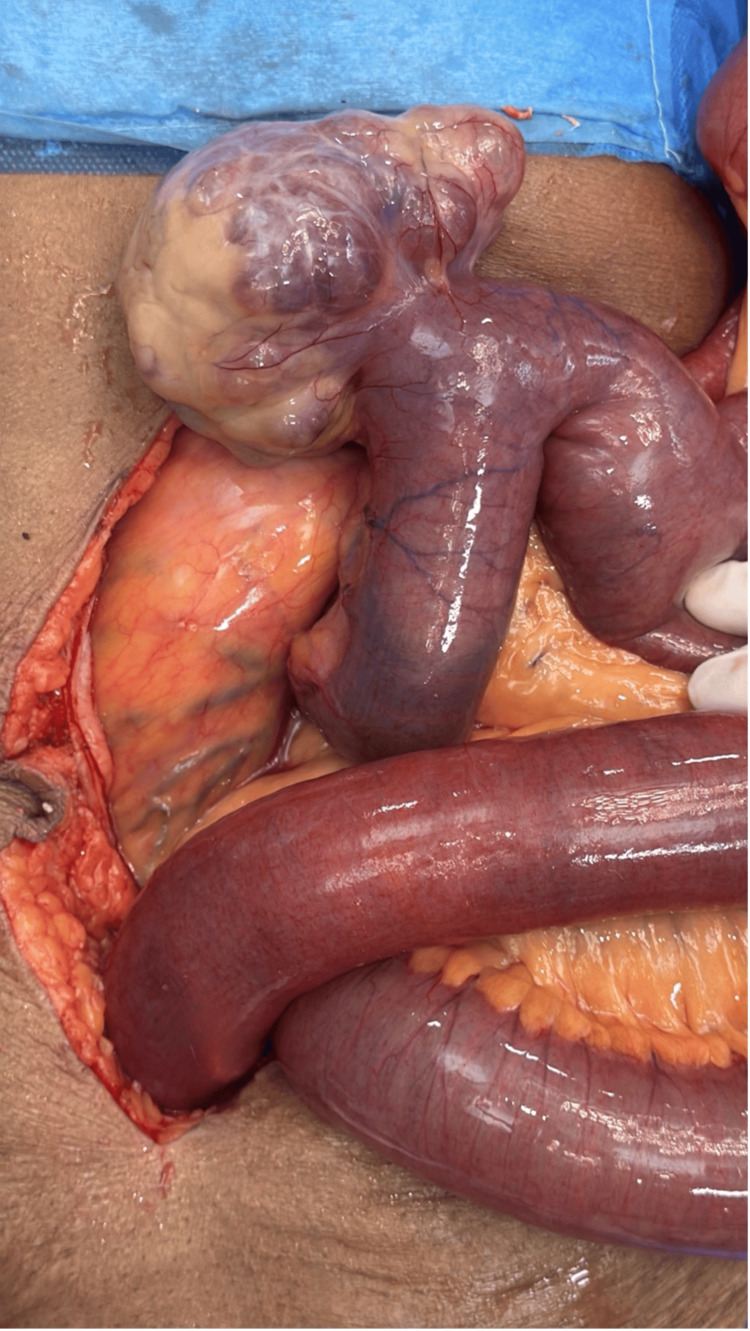
Broad-based uncomplicated jejunal diverticula

**Figure 5:**
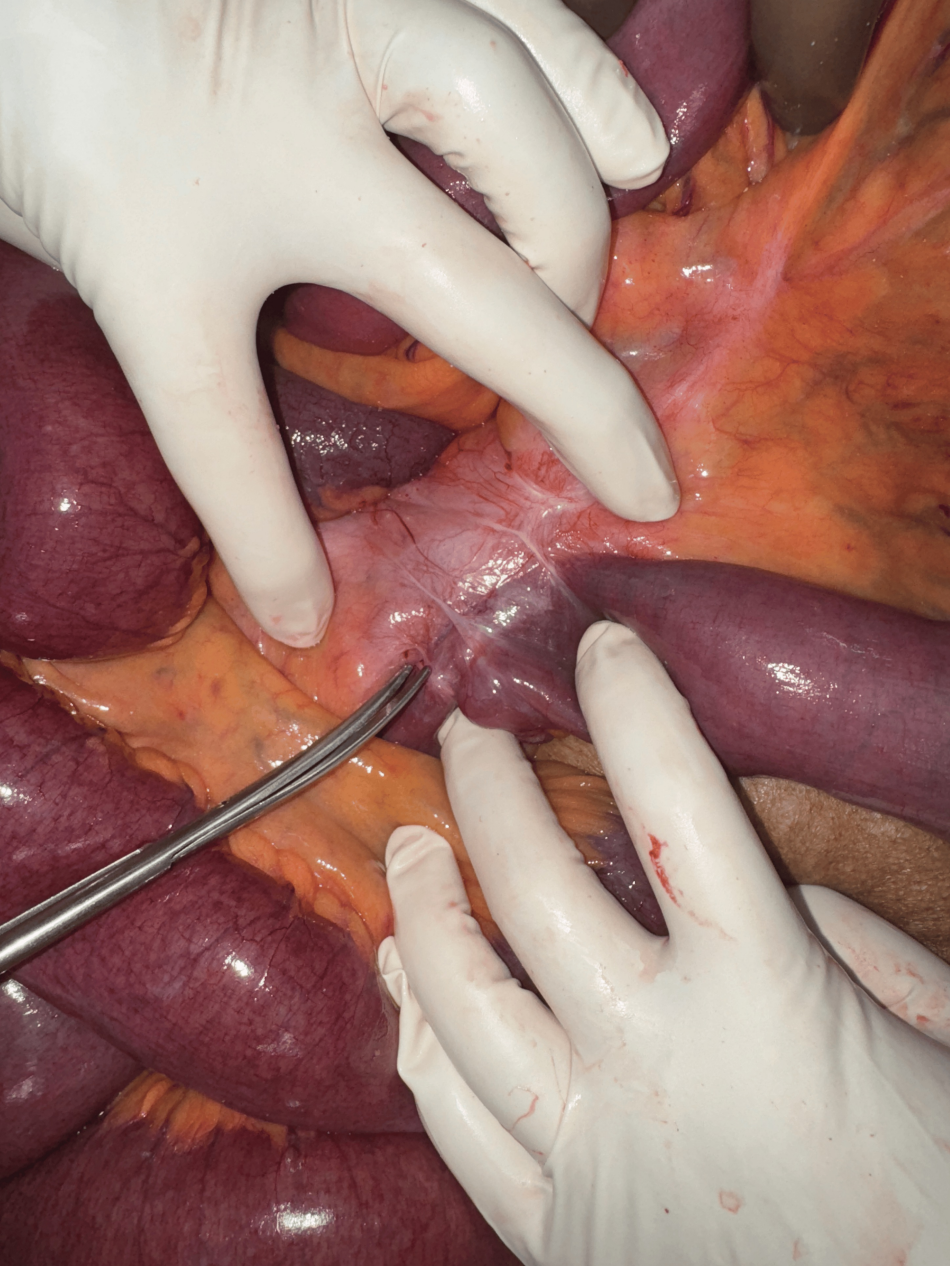
Bands noted between the small bowel and mesentery which caused the volvulus

**Figure 6:**
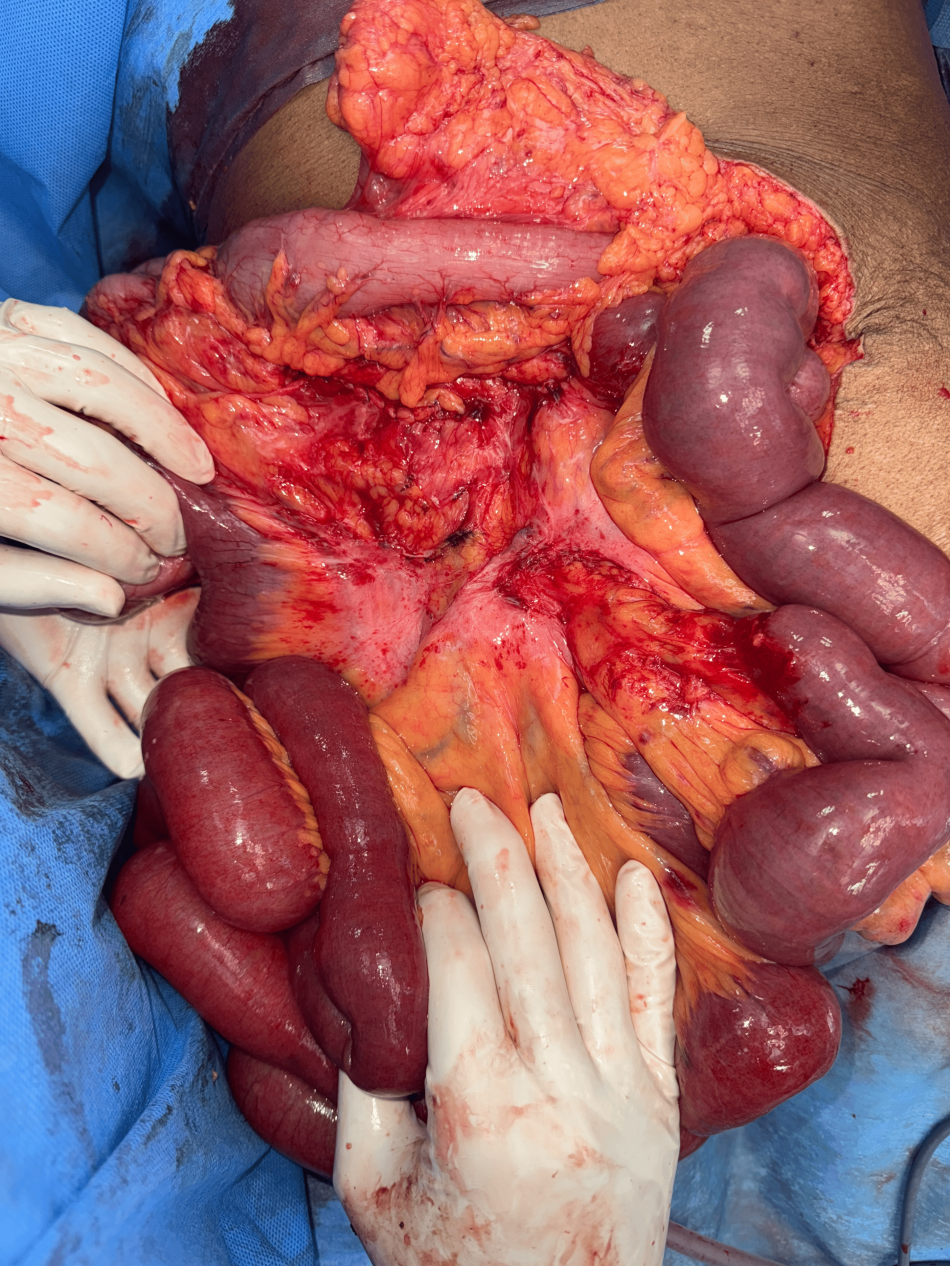
Widened mesentery and post-release state of bands and adhesions between mesentery and bowel to correct the volvulus

The cardiologist's opinion was sought for cardiac risk assessment and pacemaker mode changes. With a diagnosis of acute intestinal obstruction due to midgut malrotation with volvulus, the patient underwent emergency exploratory laparotomy following resuscitation. Intraoperatively, dilated small bowel loops and mesenteric volvulus involving the jejunal loops due to malrotation of the midgut were observed (Figure 3). An incidental finding of broad-based jejunal diverticulosis was noted in the loops of bowel involved in volvulus (Figure 4), and a 180-degree clockwise rotational abnormality of the mesentery due to Ladd's bands was identified as the cause (Figure 5). A Ladd's procedure was performed, which included the release of multiple adhesions and bands between the mesentery and small bowel to restore normal mesenteric orientation, as well as an appendectomy. The released small bowel loops and the widened mesentery were viable and demonstrated good vascularity with palpable pulsations in the mesentery (Figure 6). There were multiple jejunal diverticuli: the large ones were closer to the DJ flexure, and these were not inflamed; hence, the decision was made not to resect these. The released small bowel loops were placed to the right half of the abdomen, and the large bowel was placed to the left half of the abdomen.

Post-surgery, the patient developed a prolonged postoperative ileus. A follow-up CT scan of the abdomen on postoperative day five showed dilated bowel loops with no transition point, with normalization of the SMA-SMV axis. As the patient's condition did not improve symptomatically, an oral Gastrografin study performed on postoperative day six showed the free flow of contrast into the ileal loops. The patient passed flatus and stools post-Gastrografin study and improved symptomatically. Subsequently, she was started on oral liquids, which were gradually advanced to a soft diet. The patient was discharged on postoperative day eight. On follow-up over the next six months, she remained asymptomatic.

## Discussion

Adult midgut volvulus secondary to congenital intestinal malrotation is an uncommon but increasingly recognized cause of bowel obstruction, particularly in the elderly. Recent literature emphasizes the importance of maintaining a high index of suspicion in adults with unexplained abdominal symptoms to avoid delays in diagnosis that can result in significant morbidity or mortality due to bowel ischemia [7,9]. CT, especially contrast-enhanced studies, remains the gold standard for diagnosing midgut volvulus. The classic "whirlpool sign," indicating the twisting of the mesentery and associated vessels, remains a hallmark radiologic feature. Xiong et al. reinforced the utility of CT in diagnosing intestinal malrotation in adults, reporting over 90% diagnostic accuracy in a retrospective study spanning seven years [10].

In our case, jejunal diverticulosis coexisted with malrotation and volvulus, adding complexity to the intraoperative findings. Although jejunal diverticula are typically asymptomatic, their presence in volvulus may contribute to or exacerbate obstruction. Recent reviews by Bangeas et al. and Mansour et al. indicate a growing clinical awareness of this rare but potentially significant anatomical anomaly [11,12]. The concurrent presence of malrotation with jejunal diverticulosis is exceptionally rare, with only isolated case reports in the literature [13]. The case report by Saxena et al. described a patient with both malrotation and jejunal diverticulosis causing small bowel obstruction, though the obstruction mechanism was due to compression of the duodenum by the SMA, which differs from our case [14].

Surgical intervention remains the definitive treatment for malrotation and volvulus. Ladd's procedure, though originally developed for pediatric patients, is well-established as effective in adults. Modifications may be necessary to accommodate the anatomical changes and comorbidities commonly observed in elderly patients. Kotobi et al. emphasized timely surgical correction as a key factor in preventing irreversible bowel damage in cases of total midgut volvulus [15]. Postoperative care is equally critical. Ileus is a frequent complication following abdominal surgery in elderly individuals. Khawaja et al. have recommended early mobilization and selective use of prokinetics to mitigate postoperative ileus, a finding echoed in geriatric surgical recovery protocols [16].

Recent studies have shown that Gastrografin administration helps to safely differentiate between mechanical obstruction and functional ileus in small bowel obstruction, and its passage into the colon on follow-up imaging predicts successful non-operative management. In our patient, the administration of Gastrografin not only confirmed the absence of persistent obstruction but also contributed to the rapid resolution of ileus, allowing for an earlier return to oral intake and discharge. This aligns with growing evidence supporting Gastrografin as an effective adjunct in the postoperative management of ileus and adhesive small bowel obstruction, lowering the need for surgical re-intervention [17,18].

This report highlights the importance of early imaging, surgical vigilance, and awareness related to unusual findings, such as jejunal diverticulosis. Adult presentations of malrotation with volvulus, though rare, must not be overlooked in differential diagnoses for acute abdomen, especially given the increasing longevity and complexity among elderly surgical patients.

## Conclusions

This report illustrates the uncommon occurrence of acute midgut volvulus in an elderly patient due to congenital malrotation, with broad-based, uncomplicated jejunal diverticulosis as an incidental finding. In order to minimize surgical morbidity, the clinical management was directed by timely radiological diagnosis and the implementation of a standard Ladd's procedure, with the conscious decision to preserve asymptomatic diverticula. To ensure optimal patient outcomes, clinicians should be vigilant for unusual presentations and customize surgical intervention based on both the underlying pathology and incidental findings.
